# The carbohydrate-binding module of xylanase from *Nonomuraea flexuosa* decreases its non-productive adsorption on lignin

**DOI:** 10.1186/1754-6834-6-18

**Published:** 2013-01-30

**Authors:** Junhua Zhang, Ulla Moilanen, Ming Tang, Liisa Viikari

**Affiliations:** 1 College of Forestry, Northwest A&F University, 3 Taicheng Road, Yangling, 712100, China; 2 Department of Food and Environmental Sciences, University of Helsinki, P.O. Box 27, Helsinki, Finland

**Keywords:** Carbohydrate binding module, Xylanase, Adsorption, Xylan

## Abstract

**Background:**

The enzymatic hydrolysis step converting lignocellulosic materials into fermentable sugars is recognized as one of the major limiting steps in biomass-to-ethanol process due to the low efficiency of enzymes and their cost. Xylanases have been found to be important in the improvement of the hydrolysis of cellulose due to the close interaction of cellulose and xylan. In this work, the effects of carbohydrate-binding module (CBM family II) of the xylanase 11 from *Nonomuraea flexuosa* (Nf Xyn11) on the adsorption and hydrolytic efficiency toward isolated xylan and lignocellulosic materials were investigated.

**Results:**

The intact family 11 xylanase of *N. flexuosa* clearly adsorbed on wheat straw and lignin, following the Langmuir-type isotherm. The presence of the CBM in the xylanase increased the adsorption and hydrolytic efficiency on insoluble oat spelt xylan. But the presence of the CBM did not increase adsorption on pretreated wheat straw or isolated lignin. On the contrary, the CBM decreased the adsorption of the core protein to lignin containing substrates, indicating that the CBM of *N. flexuosa* xylanase did not contribute to the non-productive adsorption.

**Conclusion:**

The CBM of the *N. flexuosa* xylanase was shown to be a xylan-binding module, which had low affinity on cellulose. The CBM of the *N. flexuosa* xylanase reduced the non-specific adsorption of the core protein to lignin and showed potential for improving the hydrolysis of lignocellulosic materials to platform sugars.

## Background

Utilization of lignocellulosic materials offers great potential to reduce our dependence on fossil fuels. The enzymatic hydrolysis step converting lignocellulosic materials into fermentable sugars is recognized as one of the major limiting steps in biomass-to-ethanol process due to the recalcitrant and complex structure of the lignocellulosic substrate and the relatively high cost of enzymes. The fermentable sugars are derived from cellulose and hemicelluloses in lignocellulosic materials. To convert cellulose into glucose, three major cellulase groups are required: endoglucanases, cellobiohydrolases and β-glucosidase, which synergistically hydrolyze cellulose [1]. Xylans, the main hemicelluloses in hardwoods and annual plants, are closely associated with the cellulose fibrils, as well as lignin, and cover the fiber surfaces [2]. After pretreatment, even low amounts of residual xylan can limit the extent and efficiency of cellulose hydrolysis by cellulases, but the limitation can be overcome by addition of xylanases that solubilize xylan in the substrates [3-5]. Thus, xylanases play an important role in efficient hydrolysis of xylan-containing lignocellulosic materials.

Xylans in annual plants consist of a linear backbone of β-(1 → 4)-D-xylopyranosyl residues, substituted by α-L-arabinofuranosyl units in the positions of 2-O and/or 3-O, by 4-O-methyl-glucopyranosyl uronic acid in the position of 2-O, and/or by acetyl groups in 2-O and/or 3-O [6]. Furthermore, some of the arabinofuranosyl units may be esterified with ferulic or p-coumaric acids [7]. Endo-1,4-xylanases cleave the internal β-1,4-glycosyl bonds in the xylan main chain and produce xylo-oligosaccharides as main products. In the hydrolysis of lignocellulosic materials, addition of xylanases has been shown to significantly improve the performance of cellulases and to increase the cellulose conversion [8-10].

Most fungal cellulases and some hemicellulases studied so far have a complex modular architecture comprising a catalytic domain (CD) connected usually to the non-catalytic carbohydrate-binding module (CBM) via a flexible linker rich in either proline, threonine, and/or serine residues [11]. The CBMs are located either at the N- or C- terminal or both and are currently categorized into 64 defined families based on amino acid sequence similarities (http://www.cazy.org). Furthermore, these families have been categorized into three types based on their structure, function and ligand specificities: surface binding CBM (type A), glycan-chain binding CBM (type B) and small-sugar binding CBM (type C). Type A CBM includes members of families 1, 2a, 3, 5 and 10 and are recognized to bind on insoluble, highly crystalline cellulose and/or chitin [12].

It has been reported that CBMs play an important role in the improvement of enzymatic hydrolysis by cellulases [13-15]. Based on present data, the main contribution of the CBM to the enzymatic hydrolysis is the ability of CBM to target the catalytic domain to a specific substrate, thereby increasing the concentration of enzymes on the surface of the substrate. Recently, however, it has been shown that intact cellobiohydrolases and their core domains lacking CBM possess similar catalytic activity towards cellulose [16], and that cellobiohydrolases with and without CBM proceed along the cellulose chain with a similar speed [17].

The impacts of cellulose-binding modules in hemicellulases on the adsorption and hydrolysis of hemicelluloses have also been reported. Obviously, the close presence of hemicelluloses and cellulose in the substrates results in improved hydrolytic efficiency on hemicelluloses by enzymes containing a cellulose-binding module. Thus, the cellulose-binding module of *Trichoderma reesei* mannanase did not bind to mannan but increased the hydrolysis rate of insoluble mannan-cellulose complexes [18]. Fusion of the mannanase from *Aspergillus aculeatus* with a family I CBM from *A. niger* cellobiohydrolase B also improved the hydrolysis of NaOH-pretreated softwood pulp [19]. The adsorption and hydrolytic activity on insoluble xylan by the xylanases A and B from *Clostridium stercorarium*, was found to be increased by the presence of two family 6 and one family 9 cellulose-binding modules, respectively [20,21]. Fusion of the family 6 CBM from *C. stercorarium* xylanase to a *Bacillus halodurans* xylanase also resulted to an increased adsorption on cellulose and insoluble xylan [22]. However, the effect of the actual xylan binding modules on the adsorption and solubilization of xylans has received only limited attention. The two N-terminal family 22 CBMs from *Thermotoga neapolitana* xylanase A were found to bind on xylan but not on cellulose. The fusion of these CBMs with a family 10 xylanase from *Bacillus halodurans* increased the adsorption on insoluble xylan, and improved the hydrolytic efficiency of insoluble xylan but not of soluble xylan [23]. It has also been reported that the family 2b xylan-binding domain 1 from *Cellulomonas fimi* xylanase D bound on xylan but not on cellulose [24] whereas the xylanase 11A from the same fungus was shown to contain two family 2b CBMs binding on both cellulose and xylan. The CBM2b-1was shown to bind specifically on xylan and the CBM2b-2 on both insoluble and soluble oat spelt xylan, but exhibited also weak affinity to insoluble cellulose [25]. The family IIb CBM of xylanase from *Streptomyces thermoviolaceus* increased the catalytic activity of a xylanase from *Thermotoga maritima* on soluble xylan, but not on insoluble xylan [26].

The family 1 cellulose binding modules of Cel7A and Cel5A of *T. reesei* have been shown to be mainly responsible for the non-specific binding of the enzymes on lignins [27]. The intact *T. reesei* Cel7A and Cel5A enzymes were found to bind more on isolated lignins than the corresponding core domains. The β-glucosidase from *T. reesei* lacking a CBM was, however, found to bind strongly on lignin-rich residues but much less on Avicel and steam pretreated spruce [28]. Limited information on the adsorption properties of xylanases on different lignin containing materials is available.

The hydrolytic pattern of the core domain of the thermostable Xyn11 *Nonomuraea flexuosa* has been previously characterized on isolated xylans and lignocellulosic substrates [29]. Based on the C-termini amino acid sequence similarities, the xylanase Xyn11A of *N. flexuosa* contains a family II CBM [30]. In this work, the family II CBM of the Xyn11 from *N. flexuosa* was characterized with respect to its adsorption on insoluble xylan, lignin and pretreated wheat straw. The role of the CBM from *N. flexuosa* xylanase in the hydrolysis of isolated xylan was evaluated and the effect of CBM in xylanase from *N. flexuosa* on non-productive adsorption on lignin was investigated. The main objective of this work was to understand the impact of CBM from *N. flexuosa* xylanases in the total hydrolysis of lignocellulosic materials for platform sugars.

## Results and discussion

### Purification of xylanases

Most of the less thermostable enzymes in the preparation of the intact xylanase with the CBM (Nf xylF), expressed in the host strain *T. reesei* were removed already by the heat treatment (60°C, pH 6.0) for 2 hours. After the heat treatment, the protein concentration decreased from 10.0 to 2.2 mg/ml. It indicated that most of the proteins of *T. reesei* in the preparation were less thermostable and were precipitated by the heat treatment. The decrease of the specific activities of β-xylosidase, β-glucosidase, endoglucanase and FPA indicated that most of these activities were removed (results not shown). As expected, the specific activity of xylanase was increased clearly, which also indicated that the Nf xylF xylanase was thermostable.

**Figure 1:**
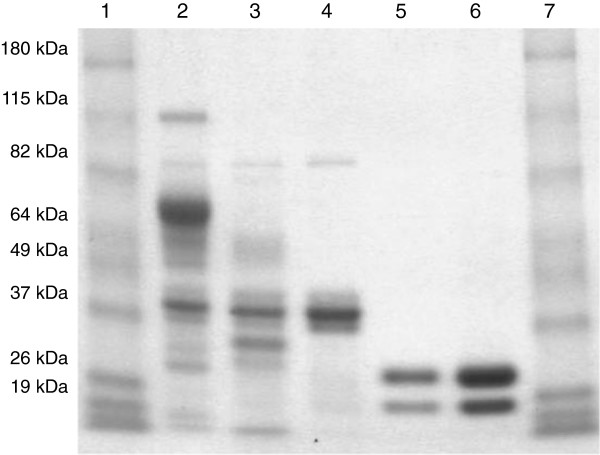
**Sodium dodecyl sulfate polyacrylamide gel electrophoresis (SDS-PAGE) of Nf xylC and Nf xylF.** (**a**) Lane 1: Molecular mass markers; Lane 2: *N. flexuosa* xylanase with CBM, original Nf xylF; Lane 3: *N. flexuosa* xylanase with CBM after heat treatment, heat-treated Nf xylF; Lane 4: Purified Nf xylF; Lane 5: Purified Nf xylC; Lane 6: *N. flexuosa* xylanase without CBM after heat treatment, heat-treated Nf xylC; Lane 7: Molecular mass markers.

The proteins were further purified by ion exchange chromatography. The purity of Nf xylF was confirmed by sodium dodecyl sulfate polyacrylamide gel electrophoresis (SDS-PAGE, Figure 1). The band at approximately 82 kDa was β-glucosidase produced by the host strain *T. reesei*. The core xylanase preparation without the CBM (Nf xylC) has been previously purified and characterized [29]. The two bands in Nf xylC corresponded to the core domain and the core domain with the linker, respectively (Figure 1), as described previously [28,29]. The molecular weights of Nf xylF and Nf xylC were 33.4 and 22.65 kDa, respectively, according to the analysis on SDS-PAGE, corresponding well to the previously analyzed values.

### Adsorption on xylan

**Figure 2:**
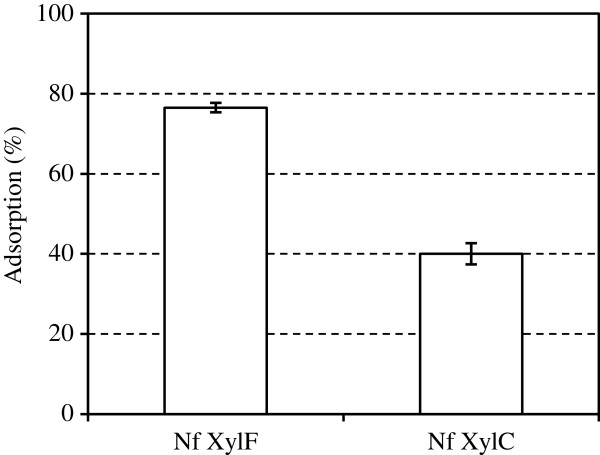
**Adsorption of Nf xylC and Nf xylF xylanase preparations on insoluble oat spelt xylan.** The ratio (%) of the amount of xylanase bound to xylan to the total amount of xylanase added into the mixture was defined as adsorption (%). Error bars represent the standard errors.

Enzyme adsorption is essential to concentrate the enzymes on the substrates in the hydrolysis of dilute substrate slurries. The adsorption of Nf xylC and Nf xylF on insoluble xylan was studied by incubating the enzymes with insoluble oat spelt xylan and determining the residual xylanase activities in the supernatants. As expected, strong adsorption (77%) on insoluble oat spelt xylan was observed by Nf xylF whereas the CBM-less Nf xylC adsorbed significantly less (40%, Figure 2). The results indicated that the CBM of the xylanase from *N. flexuosa* increased its adsorption to insoluble xylan. The results were thus in accordance with previously reported results on xylanases with cellulose or xylan binding modules [22,26].

### Hydrolysis of isolated xylans

**Figure 3:**
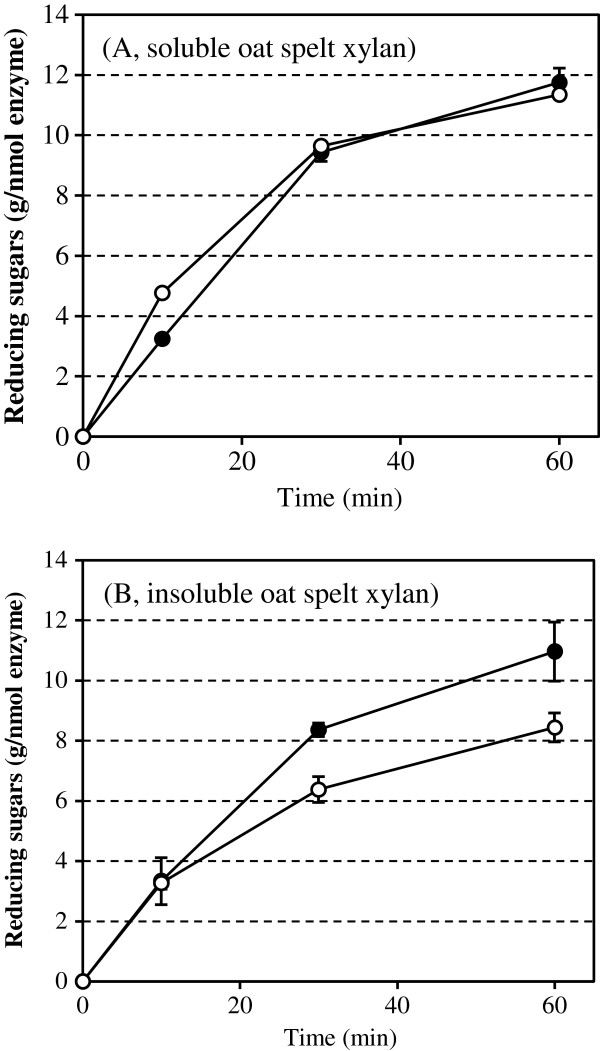
**Hydrolysis of soluble and insoluble oat spelt xylan by Nf xylC and Nf xylF.** Soluble (**A**) and insoluble oat spelt xylan (**B**) were hydrolyzed by Nf xylC (○) and Nf xylF (● ) xylanase preparations (10 nmol/g DM) at pH 5.0 and at 50ºC. Error bars represent the standard errors.

In order to compare the hydrolytic activities of Nf xylC and Nf xylF on soluble and insoluble xylan substrates, the enzymes were dosed on the same molar amount (10 nmol/g dry matter (DM)). Roughly, the same amount of reducing sugars was released from the soluble oat spelt xylan (Figure 3A), indicating that Nf xylC and Nf xylF were equally efficient in the hydrolysis of soluble xylan and the presence of CBM did not enhance the hydrolysis of soluble oat spelt xylan. More reducing sugars were, however, released from the insoluble oat spelt xylan by Nf xylF than by Nf xylC (Figure 3B), indicating that the presence of CBM improved the hydrolytic action of the Nf xylF towards insoluble xylan substrates. As previously observed, the presence of CBM obviously increased the concentration of the catalytic domain on the surface of the substrate [20,22]. As expected, a slightly lower amount of reducing sugars was released from insoluble than the soluble oat spelt xylan, especially by the Nf xylC.

### Adsorption on substrates

**Figure 4:**
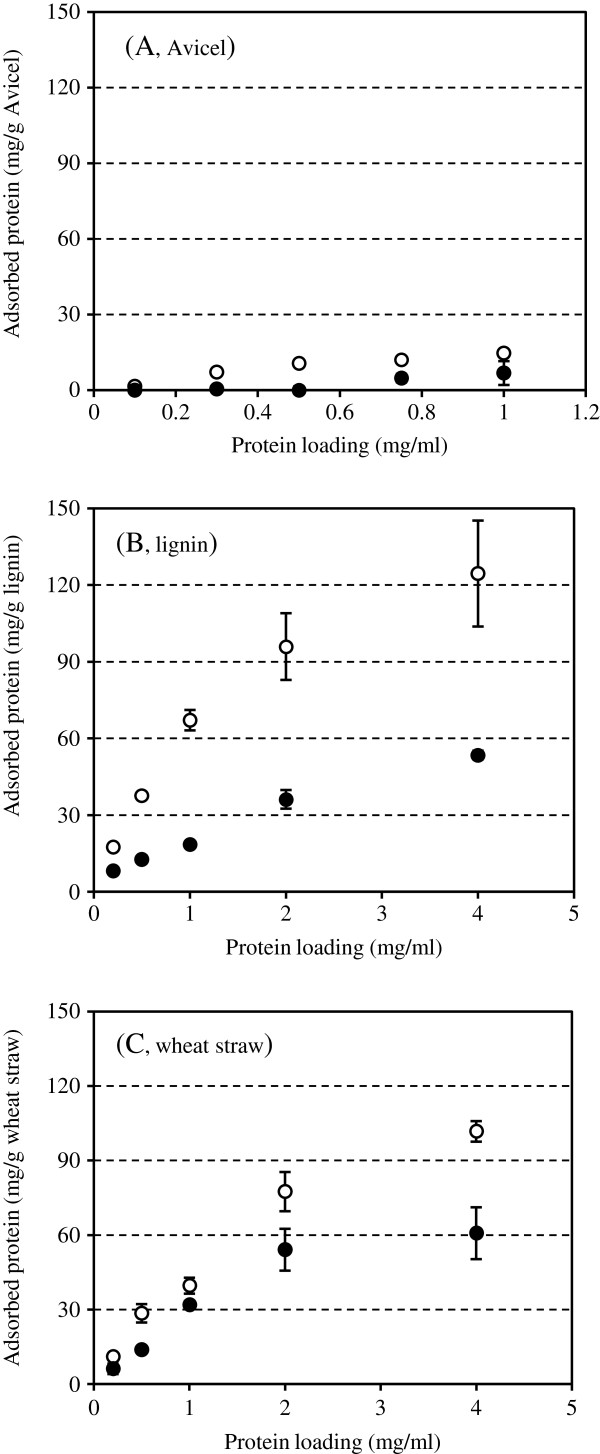
**Adsorption of Nf xylC and Nf xylF xylanase preparations on different substrates. A**: Avicel; **B**: lignin; **C**: hydrothermally pretreated wheat straw. Adsorption experiments of Nf xylC (○) and Nf xylF (● ) were performed at pH 5.0 and at 4°C. Error bars represent the standard errors.

Adsorption experiments showed that both Nf xylC and Nf xylF had low affinity and hardly adsorbed on Avicel (Figure 4A). The CBM of Nf xylF had adsorption properties similar to the family IIb xylan-binding domain of the xylanase from *C. fimi*, which bound on both soluble and insoluble xylans but did not bind on cellulose [24]. The results thus suggested that the native CBM of the Nf xylF was truly a xylan-binding domain, obviously important in the hydrolysis of insoluble xylans.

Langmuir isotherm has been used to describe the adsorption of cellulases on cellulose, lignin or lignocellulosic materials [31-34]. The adsorption of Nf xylC and Nf xylF on lignin and wheat straw were investigated (Figure 4), and the adsorption parameters including maximum adsorption capacity (*P*_ads,m_), affinity constant (*K*_p_) and strength of binding (*A*) were calculated (Table 1). The results showed that the adsorption of Nf xylC and Nf xylF on lignin and wheat straw followed well the Langmuir adsorption isotherms with correlation coefficients of R^2^ > 0.85. The maximum adsorption capacities of the CBM-less Nf xylC on lignin (135.8 mg/g substrate) and wheat straw (138.1 mg/g substrate) were higher than those of the intact Nf xylF, surprisingly indicating that the Nf xylF bound less on lignin than the Nf xylC. The maximum adsorption capacities of Nf xylC and Nf xylF on wheat straw were about equal to those on lignin, which could be due to the high content of lignin (42.7%) in pretreated wheat straw. The presence of CBM of xylanase from *N. flexuosa* decreased the adsorption on lignin (Figure 4), resulting in the lower maximum adsorption capacities of Nf xylF than those of Nf xylC. The lower maximum adsorption capacity (77.9 mg/g substrate) was observed for Nf xylF on lignin than on wheat straw possibly due to its relatively lower lignin content. The highest affinity constant (3.31 ml/mg) and strength of binding (449.5 ml/g substrate) were observed for the core enzyme Nf xylC on lignin. The results clearly revealed that the CBM-less Nf xylC had higher affinity for lignin and wheat straw than the intact Nf xylF. Previously, the adsorption of xylanase on corn stover substrates obtained by different pretreatments has been investigated [33] and the maximum adsorption capacity for lime-pretreated corn stover was close to the values of Nf xylC for lignin and wheat straw, and that for dilute acid-pretreated corn stover was clearly lower.

**Table 1:** Adsorption parameters of Nf xylC and Nf xylF for lignin and wheat straw

**Solid**	**Enzyme**	**Maximum Adsorption capacity, *P***_**ads,m **_**(mg/g substrate)**	**Affinity constants,*K***_**p **_**(ml/mg)**	**Strength of binding, R = *P***_**ads,m **_**× *K***_**p **_**(ml/g substrate)**	**R**^**2**^
Lignin	Nf xylC	135.8	3.31	449.50	0.99
	Nf xylF	77.9	0.56	43.62	0.85
Wheat straw	Nf xylC	138.1	0.92	127.05	0.96
	Nf xylF	98.06	0.56	54.91	0.89

The results presented here thus revealed that the xylanase from *N. flexuosa* clearly adsorbed on wheat straw and lignin non-specifically by the core domain, and the native CBM did not increase the adsorption on lignocellulosic materials or lignin. Adsorption experiments on Avicel cellulose showed that either the Nf xylC or the Nf xylF were hardly bound on Avicel, as compared to lignin and wheat straw. The results also indicated that the CBM of *N. flexuosa* xylanase did not contribute to the non-productive adsorption of the enzyme on lignin but on the contrary, the CBM of *N. flexuosa* xylanase seemed to physically prevent the strong binding of the core protein on lignin. Thus, the CBM obviously recognizes and adsorbs to the xylan substrate but seems to reduce the adsorption of the core protein to lignin.

## Conclusions

The CBM of the Nf Xyn11 clearly improved the hydrolytic efficiency of the enzyme towards insoluble xylan by concentrating the enzyme on the substrate. The presence of the CBM in xylanase was, however, not shown to increase the adsorption on lignin or lignocellulosic materials. In order to improve the enzymatic conversion processes of lignocellulosic materials, use of xylanases with the CBM would thus be more beneficial than xylanases without the CBM.

## Methods

### Substrates and chemicals

Oat spelt xylan was obtained from Serva (Heidelberg, Germany). The lignin preparation was produced by extensive enzymatic removal of carbohydrates from thermochemically pretreated spruce followed by protease treatment to remove the bound enzymes [35]. Hydrothermally pretreated wheat straw was a kind gift of Inbicon (Fredericia, Denmark). In order to reduce the effect of dissolved sugars in the substrate, the pretreated wheat straw was washed three times by deionized water and the sugar composition was determined after acid hydrolysis by high performance liquid chromatography [36] using an analytical CarboPac PA-1 column (Dionex Corp., Sunnyvale, CA, USA), as described in the NREL procedure [37]. The washed pretreated wheat straw contained 58.9% glucan, 3.2% xylan and 42.7% lignin. D-Xylose and D-glucose (Merck, Darmstadt, Germany) were used as carbohydrate standards. All other chemicals used were of analytical grade and purchased from Sigma or Merck.

Soluble and insoluble oat spelt arabinoxylans were prepared by using a modified method of Ryan *et al*. [38]. Oat spelt xylan (4 g) was suspended in 400 mL of distilled water and stirred overnight at room temperature. The insoluble fraction was recovered by centrifugation at 10 000 × g for 20 min and at 4°C. The insoluble fraction was washed several times with Milli-Q water (Milli-Q Plus; Millipore, Billerica, MA, USA). After that, the sediment was lyophilized and used as insoluble oat spelt xylan for hydrolysis. The soluble fraction was lyophilized and used as soluble oat spelt xylan.

### Enzymes

The xylanases with and without CBM from *N. flexuosa* were heterologously produced in a *T. reesei* strain where the genes *cbh1*, *cbh2*, *egl1* and *egl2*, encoding for cellobiohydrolase I, cellobiohydrolase II, endoglucanase I and endoglucanase II, respectively, had been deleted according to the method described elsewhere [28,39]. All these enzyme preparations were kindly provided by Roal Oy (Rajamäki, Finland).

### Enzyme purification

To remove the less thermostable enzymes produced by the host strain *T. reesei*, the two xylanase preparations with and without CBM were adjusted to pH 6.0 and were treated at 60°C for 2 hours. In the heat treatment, the less thermostable enzymes in the xylanase preparations were removed (Figure 1). The heat-treated xylanase preparation Nf xylC was desalted by ultrafiltration through an Amicon membrane with a 10 kDa molecular mass cut-off (Amicon, USA) and purified by ion-exchange chromatography. The column with DEAE-Sepharose Fast Flow (2.6 × 13 cm; Amersham Biosciences, Uppsala, Sweden) was equilibrated with 20 mM Tris-HCl buffer at pH 8.0. Elution of bound protein from the column was accomplished by a linear gradient of 20 mM Tris-HCl buffer and 20 mM Tris-HCl buffer containing 0.5 M NaCl. During the purification, the main peak with xylanase activity was collected for the adsorption and hydrolysis experiments. For the purification of the *N. flexuosa* xylanase with CBM (Nf xylF), the same method and system was used but the pH of buffer was adjusted to pH 9.1. At pH 8.0, the Nf xylF did not bind to the column. During the purification, two main peaks were obtained and the peak with high xylanase activity was collected for the adsorption and hydrolysis experiments. After purification by ion-exchange chromatography, hydrophobic interaction chromatography with Phenyl Sepharose Fast Flow (Amersham Biosciences, Uppsala, Sweden) was applied for further purification but the band of approximately 82 kDa could not be removed.

### Enzyme analysis

Xylanase activity was assayed using 1% (w/v) birchwood xylan (Roth 7500, Karlsruhe, Germany) as a substrate in 50 mM sodium citrate buffer according to the method of Bailey *et al*. [40]. The assay was performed at pH 5.0 and 50°C for 5 minutes. The amount of reducing sugars liberated was determined using the dinitrosalicylic acid method with xylose used as standard [41]. One katal (1 kat) of the enzyme activity is defined as the amount of enzyme that catalyzes the release of 1 mole of reducing sugar per second. All activities presented are average values of three separate determinations.

Protein was quantified by the Lowry method, using bovine serum albumin (Sigma Chemical Co., USA) as standard [42]. SDS-PAGE was performed on 12% polyacrylamide gel using the method of Laemmli [43]. A pre-stained protein ladder (Invitrogen, Carlsbad, CA, USA) was used as a molecular weight standard. After electrophoresis, the gel was stained with Coomassie brilliant blue G-250 (Bio-Rad, Hercules, CA, USA).

### Adsorption experiments

The insoluble oat spelt xylan was used for xylanase adsorption experiments. The xylanase preparations (2 mg protein/g DM) were incubated with 1% (w/v) xylan at 4°C for 1 h. After centrifugation the residual xylanase activity in the supernatant was measured. The amount of enzyme bound to xylan was estimated from the difference between the xylanase activities in the supernatant before and after incubation. In addition to xylan, Avicel, lignin prepared from spruce, and hydrothermally pretreated wheat straw were also used for adsorption studies. The experiments were carried out in 50 mM sodium citric acid buffer (pH 5.0) in 1% Avicel or lignin or wheat straw consistency at 1.5 ml volume. The samples were incubated with 10–400 mg/g DM of xylanase preparation for 90 min at 4ºC with magnetic stirring. After this the solids and liquids were separated by centrifugation at 4ºC (10 000 × g, 10 min). The protein adsorbed was measured by subtracting the protein in supernatant from the total protein loaded. All adsorption experiments were done in triplicates and average values are presented.

### Calculation of adsorption parameters

Adsorption parameters were calculated according the reported method [44,45] using the Langmuir-type adsorption isotherm as Equation below:

$$p_{\text{ads}}=\frac{K_{p}P_{\text{ads},m}}{1+K_{p}P}\cdot P$$

where *P*_ads_ is the amount of adsorbed enzyme (mg enzyme/g substrate); *P* is the amount of free enzyme (mg enzyme/ml); *P*_ads,m_ is the maximum adsorption capacity (mg enzyme/g solid); *K*_p_ is the adsorption equilibrium constant (ml/mg enzyme) and is a measurement for the adsorption affinity. *P*_ads,m_ and *K*_p_ can be calculated from the plots of *P*/*P*_ads_ vs. *P*, which gave fairly good straight lines. The adsorption strength of the enzyme *A* is calculated from *P*_ads,m_ and *K*_p_ (*A* = *P*_ads,m_ × *K*_p_).

### Hydrolysis of isolated xylan

The hydrolysis of soluble and insoluble oat spelt xylan (2.5 mg/mL) was carried out in test tubes with a working volume of 2 mL. The enzyme dosage was 10 nmol/g DM, based on the molecular weights of the cloned enzymes. The hydrolysis of xylan substrates was carried out in 50 mM sodium citrate buffer at pH 5.0 and at 50°C. Aliquots were removed periodically at different time intervals and boiled for 10 minutes to stop the enzymatic hydrolysis. Two replicates were carried out, and average values of reducing sugars are presented.

## Abbreviations

CBM: Carbohydrate binding module; CD: Catalytic domain; DM: Dry matter; Nf xylC: Xylanase without CBM from *N. flexuosa*; Nf xylF: Xylanase with CBM from *N. flexuosa*; SDS-PAGE: Sodium dodecyl sulfate polyacrylamide gel electrophoresis.

## Competing interests

The authors declare that they have no competing interests.

## Authors’ contributions

JZ carried out the experimental enzyme work, analyzed the results and drafted the manuscript. UM prepared the lignin and participated in the planning of the adsorption experiments and in the preparation of the manuscript. MT reviewed the paper. LV designed and coordinated the overall study and finalized the paper. All authors approved the final manuscript.
